# Privacy-Preserving Location-Based Query Using Location Indexes and Parallel Searching in Distributed Networks

**DOI:** 10.1155/2014/751845

**Published:** 2014-03-25

**Authors:** Cheng Zhong, Lei Liu, Jing Zhao

**Affiliations:** School of Computer and Electronics and Information, Guangxi University, Nanning, Guangxi 530004, China

## Abstract

An efficient location-based query algorithm of protecting the privacy of the user in the distributed networks is given. This algorithm utilizes the location indexes of the users and multiple parallel threads to search and select quickly all the candidate anonymous sets with more users and their location information with more uniform distribution to accelerate the execution of the temporal-spatial anonymous operations, and it allows the users to configure their custom-made privacy-preserving location query requests. The simulated experiment results show that the proposed algorithm can offer simultaneously the location query services for more users and improve the performance of the anonymous server and satisfy the anonymous location requests of the users.

## 1. Introduction

Recently, with the development of the mobile wireless communication location technology, the location-based service is emerged. The location information of the users is made of their identifiers and temporal and spatial information [1]. Another important problem related to the location information services is to preserve the location privacy of the user [2]. Using the anonymity on the location-based services [3] is a direct and effective method to prevent the quasi identifiers of the users. Gruteser et al. [4] introduced the* k*-anonymity model to investigate the problem of preserving the location privacy of the users. Kido et al. [5] used the dummies to study the anonymous communication technique for the location-based services.

One of the key issues for the privacy-preserving location-based services in the distributed networks is to balance the quality of query services and the privacy protection of the users. In this paper, we will propose an efficient privacy-preserving location-based query algorithm using parallel searching to improve the efficiency of the anonymous server, which not only can protect the location privacy of the user but also obtain the location query services. The remainder of this paper is organized as follows. In Section 2, we give the related work about the privacy-preserving location-based techniques. In Section 3, we propose an efficient privacy-preserving location-based query algorithm using location indexes and parallel searching in the distributed networks.  Section 4 reports the simulated experimental results.  Section 5 concludes the paper.

## 2. Related Work

By applying the personalized* k*-anonymity model, Gedik and Liu [6] proposed the architecture and the algorithms to protect the location privacy of the user. Chow et al. [7] proposed a distributed* k*-anonymity model and a peer-to-peer spatial cloaking algorithm for the anonymous location-based services. Ghinita et al. [8] investigated the anonymous location-based query method in the distributed mobile systems. By using the distributed hash table to select the anonymous set of the users, Ghinita et al. [9] implemented the anonymous location-based query services in the mobile P2P system. Zhong and Hengartner [10] used the secure multiparty computation protocol to design a distributed* k*-anonymity protocol for protecting the location privacy. By using the obfuscation method and vague location information of the user, Duckham and Kulik [11] presented a privacy-preserving location query algorithm. Mokbel [12] proposed a location-obfuscation method which allows the server to record the real identifier of the user but decreases the precision of the location information to protect the location privacy. By introducing the trusted third party, Mokbel et al. [13] proposed a location service query method without compromising privacy.

By using the space transformation, Khoshgozaran and Shahabi [14] gave a blind evaluation of the nearest neighbor query to protect the location privacy. Ghinita et al. [15] studied the private query method in the location-based services by partitioning the space into several areas and mapping these areas into the points in Hilbert curve. Pietro and Viejo [16] developed a probabilistic and scalable protocol which guarantees the location privacy of the sensors replying to the query. Raj et al. [17] proposed a realistic semiglobal eavesdropping attack model and showed its effectiveness in compromising an existing source-location preserving technique and designed a new protocol which preserves *α*-angle anonymity by adapting the conventional function of data mules. Zhao et al. [18] developed the optimal solutions to some special cases through dynamic programming and several heuristics for the general case to the location privacy-preserving problem. Pingley et al. [19] implemented a context-aware privacy-preserving location-based services system with integrated protection for both data privacy and communication anonymity and integrated it with Google Maps. Tan [20] proposed a conditional privacy-preserving authentication and access control scheme for the pervasive computing environments, in which the registration servers and authentication servers do not need to maintain any sensitive verification tables. Xi et al. [21] showed that the privacy-preserving shortest path routing problem can be solved with the private information retrieval techniques without disclosing the origin or the destination.

By introducing local suppression to trajectory data anonymization to enhance the resulting data utility, Chen et al. [22] obtained a (*K*, *C*)_*L*_-privacy model on trajectory data without paying extra utility and computation cost and proposed an anonymization framework that is independent of the underlying data utility metrics and is suitable for different trajectory data mining workloads. Based on extending the private equality primitive, Buchanan et al. [23] presented a novel encryption method for preserving the location and trajectory path of a user by privacy-enhancing technologies, which has significant improvement in the computation speed. Cicek et al. [24] grouped the points of interest to create obfuscation areas around sensitive locations and used the map anonymization as a model to anonymize the trajectories and proposed a new privacy metric *p*-confidentiality that ensures location diversity by bounding the probability of a user visiting a sensitive location with the *p* input parameters. Li and Jung [25] proposed a fine-grained privacy-preserving location query protocol (PLQL) to solve the privacy issues in existing LBS applications and provide various location-based queries. The protocol PLQL can implement semifunctional encryption by novel distance computation and comparison protocol and support multilevel access control. Dewri and Thurimella [26] proposed a user-centric location-based service architecture, that the users can observe the impact of location inaccuracy on the service accuracy, and constructed a local search application and demonstrated how the meaningful information can be exchanged between the user and the service provider to allow the inference of contours depicting the change in the query results across a geographic area.

## 3. Privacy-Preserving Location-Based Services System and Algorithm

### 3.1. Anonymous Location Query Services System

**Figure 1:**
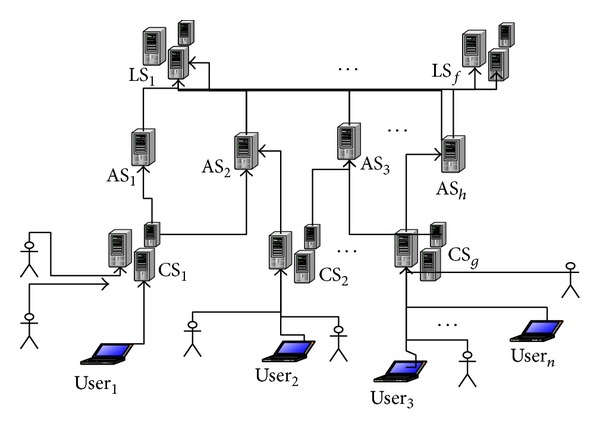
Location-based services system with the anonymous server.

The privacy-preserving location-based services system in the distributed networks includes mobile users, communication services providers CS, and location service providers LS, in which the independent trusted third party will provide the anonymous servers AS [6], which is described in Figure 1.

The anonymous location-based query process is as follows.

**Step 1**

The users acquire their locations (*x*, *y*, *r*) via the communication services provider, where *x* and *y* are the two-dimensional location coordinates of the users, respectively, and *r* represents the location precision.

**Step 2**

The users send the service request information (Uid, (*x*, *y*, *r*), profile(*A*_min⁡_, *A*_max⁡_, *K*_*s*_, *K*_*t*_, *t*, Pre), Cont) to the anonymous server, where Uid is the identifier of the user, (*x*, *y*, *r*) is the current location information of the user, profile (*A*_min⁡_, *A*_max⁡_, *K*_*s*_, *K*_*t*_, *t*, Pre) represents the configuration file of the users, *A*_min⁡_ and *A*_max⁡_ denote the minimum and maximum requirements for anonymous areas, *K*_*s*_ and *K*_*t*_ are the temporal and spatial anonymous requests, respectively, *t* is the service time demand, Pre represents the set to the anonymity priority or services priority, and Cont is the content of the query.

**Step 3**

The anonymous server receives the request from the user, generates the anonymous sets, and sends the information ((*X*, *Y*, *R*), (zid_1_, Cont_1_),…, (zid_*k*_, Cont_*k*_)) to the location service server, where (*X*, *Y*, *R*) is the anonymous area and zid_*i*_ is the *i*th anonymous identifier of the user and Cont_*i*_ represents the content of the *i*th request from the user, *i* = 1 ~ *k*.

**Step 4**

The location service server receives the requests of the user and returns the processed results (zid_1_, result_1_),…, (zid_*k*_, result_*k*_) to the anonymous server, and the anonymous server sends the transformed ID result to the user.

### 3.2. Privacy-Preserving Location Query Algorithm

Assume that the *k* users want to request location-based query services and the* i*th anonymous request is *k*_*i*_, *k* ≥ max⁡{*k*_*i*_}. If the users are evenly distributed in the space range, the probability that their request information can be guessed will be 1/*k* and the probability that the actual locations of the users can be guessed will be 1/(*πR* ^2^), respectively. We know the more the users in the space range, the more the anonymous requests and the larger the generated anonymous area, the better the anonymous effect. But the computational cost to search the anonymous space will increase, and the quality of obtained location-based services may be relatively poor. The multiple searching threads are executed in parallel to accelerate the generation of the candidate anonymous set for each request queue and compute the density *ρ* = *k*/(*πR* ^2^) of the user for all the candidate anonymous sets and the distribution of the users in the anonymous sets *C* = |(*N*_1_ + *N*_2_)−(*N*_3_ + *N*_4_)| + |(*N*_1_ + *N*_4_)−(*N*_3_ + *N*_2_)|, where *N*_*j*_ is the number of the users in the *j*th quadrant among the four partitioned quadrants, *j* = 1 ~ 4.

When the location anonymous server has received the request from the user, it searches the location indexes in B-tree and inserts the location information into the request queue. If it is necessary to establish a new request queue, the location indexes will be updated. The multiple threads search in parallel and select quickly the anonymous areas in the request queues. The anonymous server handles the selected anonymous areas and provides the appropriate location services for the users.

To establish the bidirectional indexes, each element in the request queues is arranged into the form (Uid, 〈*x*, *y*, *r*〉, 〈*R*_min⁡_, *R*_max⁡_, *k*_*s*_, *t*, *k*_*t*_, pre〉, Cont, *next, *pre). The performance of the anonymous server is directly affected by the number of the request queues on the anonymous server. We assume that there are *n* location query requests and *n* request queues on the anonymous server; the *n* location query requests are evenly distributed in the range with area *S* and the maximum anonymous radius *R*, and the number of the request queues is *S*/(*πR* ^2^). B-tree is used to construct the location indexes with the directions *X* and *Y* on the anonymous server. The two main algorithms running on the anonymous server are the Request Enqueue Algorithm and Anonymous Set Generation Algorithm, which are described as follows.

**Algorithm 1**

Request Enqueue Algorithm.

Begin

1. The request (Uid, 〈*x*, *y*, *r*〉, 〈*R*_min⁡_, *R*_max⁡_, *k*_*s*_, *t*, *k*_*t*_, pre〉, Cont) is received and it is expanded to (Uid, 〈*x*, *y*, *r*〉, 〈*R*_min⁡_, *R*_max⁡_, *k*_*s*_, *t*, *k*_*t*_, pre〉, Cont, *next, *pre).
2. B-tree indexes with the condition |*X* − *x*| < 2*R*_max⁡_ along the direction *X* is searched.
  (2.1) If the searching in the direction *X* is unsuccessful, the request is inserted into the queue, the indexes in the direction *X* are updated, and the indexes in the direction *Y* are added.
  (2.2) If the searching in the direction *X* is successful, B-tree index with the condition |*Y* − *y*| < 2*R*_max⁡_ along the direction *Y* is searched.
    (2.2.1) If the searching in the direction *Y* is unsuccessful, the current request is inserted into the request queue and the indexes in the direction *Y* are updated.
    (2.2.2) If the searching in the direction *Y* is successful, the current request is inserted into the request queue in the chronological order.

End.

**Algorithm 2**

Anonymous Set Generation Algorithm.

Begin

1. The temporal-spatial queue *L*_*T*_ is constructed, which each element in *L*_*T*_ links a request queue.
2. The Request Enqueue Algorithm is executed to generate a new request queue (Uid, 〈*x*, *y*, *r*〉, 〈*R*_min⁡_, *R*_max⁡_, *k*_*s*_, *t*, *k*_*t*_, pre〉, Content, *next, *pre) and this request queue is inserted into queue *L*_*T*_ in the chronological order, where *s* and *t* represent the space and time respectively.
3. Each element in queue *L*_*T*_ is searched, and multiple threads are generated according to the condition |*T* − *T*_*s*_| < *δ*, where *T* is the time when the element *L* in queue *L*_*T*_ wants to generate the request queue, *T*_*s*_ is the current time of the running system, and *δ* is the threshold. Each request queue is assigned to a thread.
4. Multiple threads are run in parallel, and each thread is responsible for the following operations.
  (4.1) If the number of the elements in the request queue is smaller than *k*, the elements which satisfy the condition |*T* − *T*_*s*_| < *δ* are searched and those elements with priority pre are deleted to form request queue set *Ω*. When *Ω* is not empty, the density *ρ* of the user is queried by the communication service provider, anonymous area *A* and radius *R*_*xm*_ with the minimum anonymous request are computed, and the anonymous request set is generated by the radius *R*_*xm*_ and the centroid of all elements in set *Ω*. The ID disturbing algorithm is executed to disturb the ID of the user and the anonymous request set is submitted to the location services server.
  (4.2) If the number of the elements in the request queue is larger than *k*, *m* threads are generated, where *m* is the number of elements in the request queue. Each thread executes the following operations.
    (4.2.1) If the three points (*x*_1_, *y*_1_), (*x*_2_, *y*_2_) and (*x*_3_, *y*_3_) in the anonymous range are located in a straight line, the coordinate of the center in the anonymous request set is (*x*_0_ = (*x*_1_ + *x*_2_ + *x*_3_)/3,  *y*_0_ = (*y*_1_ + *y*_2_ + *y*_3_)/3); if not, the center of the circum of the triangle with coordinates (*x*_1_, *y*_1_), (*x*_2_, *y*_2_) and (*x*_3_, *y*_3_) is the center in the anonymous request set.
    (4.2.2) If *R*_*xm*_ ≤ min⁡{*R*_max⁡_}, a candidate anonymous area with circle center (*x*_0_, *y*_0_) and radius min⁡{*R*_max⁡_} is generated.
    (4.2.3) Number *s* of the elements in the circle is computed, and the farthest point from the circle center and its distance *D*_max⁡_ are recorded. If *s* < *k*, then report failure.
    (4.2.4) If the number of the elements which satisfy the anonymous request is also smaller than *k*, then report failure.
    (4.2.5) If *x*_*j*_ − *x*_0_ > 0 and *y*_*j*_ − *y*_0_ > 0 then *N*_1_ = *N*_1_ + 1, if *x*_*j*_ − *x*_0_ < 0 and *y*_*j*_ − *y*_0_ > 0 then *N*_2_ = *N*_2_ + 1, if *x*_*j*_ − *x*_0_ < 0 and *y*_*j*_ − *y*_0_ < 0 then *N*_3_ = *N*_3_ + 1, and if *x*_*j*_ − *x*_0_ > 0 and *y*_*j*_ − *y*_0_ < 0 then *N*_4_ = *N*_4_ + 1, *j* = 1 ~ 3.
    (4.2.6) If each element within the circle satisfies the anonymous request, then Δ = min⁡{*R*_max⁡_} − *D*_max⁡_ is computed. If Δ goes beyond the threshold, the radius of the circle is reduced until Δ is in the threshold. Finally, the new radius *R*_0_ is obtained.
    (4.2.7) The anonymous area *A*((*x*_0_, *y*_0_), *R*_0_), the set *Q* including all the request elements in this area and number *N*_*Q*_ of the elements in set *Q* are returned, and the density *ρ* = *N*_*Q*_/(*πR*_0_^2^) of the users in the anonymous set is computed.
  (4.3) The ID disturbing algorithm is executed to disturb the ID of the user, set *Q* is submitted to the location service server, and queue *L*_*T*_ is renewed by the elements which are not in set *Q* and the location indexes are updated.

End.

## 4. Experiment

We used a multicore computer to simulate the anonymous server and the PC computers to simulate the users to request concurrently the location services. Redhat 5.1 and MySQL 5.5 are run on the anonymous server, respectively, and Ubuntu 10.04 is run on the clients. The presented algorithms are implemented by Java programming with JDK7.0 and socket communication.

The Thomas Brinkhoff road network data generator is applied to produce the location service requests, and the OldenBurg urban communication network information is used as the input data of the road network data generator. The anonymous server deals with the location query and the anonymous requests from the users. The value of pre is set to service priority. The values of the relative experimental parameters are listed in Table 1.

**Figure 2:**
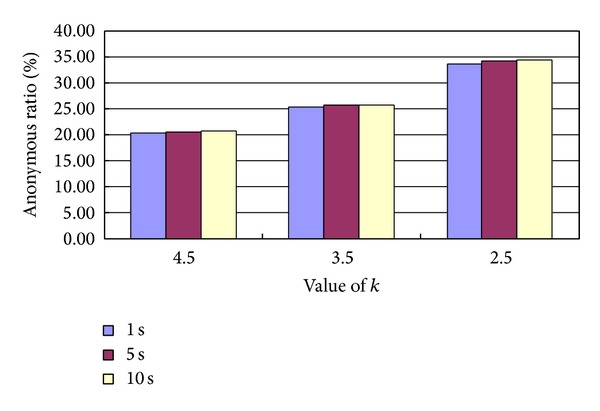
Ratio of the temporal-spatial anonymity with different waiting time and average anonymous request.

We first test that the waiting time of the user and the average anonymous value of *k* are how to impact the ratio of the temporal-spatial anonymity. The obtained simulation experimental result is given in Figure 2.

From Figure 2, we can see that the longer the waiting time of the user, the higher the ratio of the temporal-spatial anonymity and the smaller the average anonymous request, the higher the ratio of the temporal-spatial anonymity.

**Figure 3:**
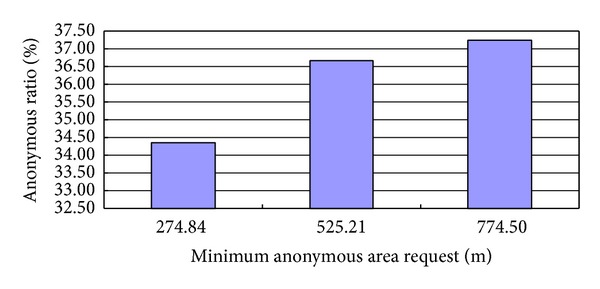
Ratio of the temporal-spatial anonymity with different anonymous space requests.

The result in Figure 3 shows that the larger the anonymous space request, the higher the ratio of the temporal-spatial anonymity and the ratio of the temporal-spatial anonymity changes significantly along with the increase of the anonymous space request. This illustrates that the different anonymous space requests will affect remarkably the ratio of the temporal-spatial anonymity.

**Figure 4:**
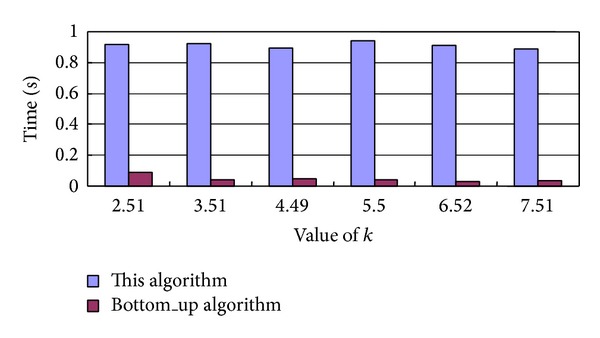
Required processing time for our algorithm and the Bottom_up algorithm.

**Figure 5:**
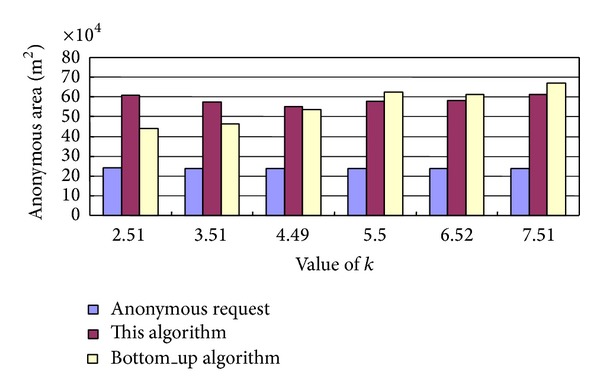
Anonymous area of our algorithm and the Bottom_up algorithm.

The required processing time and the anonymous area about our algorithm and the Bottom_up algorithm [13] are shown in Figures 4 and 5, respectively, where the minimum anonymous range partitioned some small square areas with a length of 300 m of a side and 3 users are initially contained within the minimum anonymous range.

We can see form Figure 4 that the required processing time for the Bottom_up algorithm is much less than the required time for our algorithm. This is because our algorithm wants to process more location service requests than the Bottom_up algorithm in order to achieve better privacy-preserving effect.

The results in Figure 5 show that along with the increase of the value of *k*, the anonymous area for the Bottom_up algorithm is increased, but the anonymous area for our algorithm is relatively stable; when the value of *k* is larger than 5.5, the anonymous area for our algorithm is smaller and the quality of the anonymous location service is better; in other words, the degree of privacy protection for our algorithm is higher.

**Figure 6:**
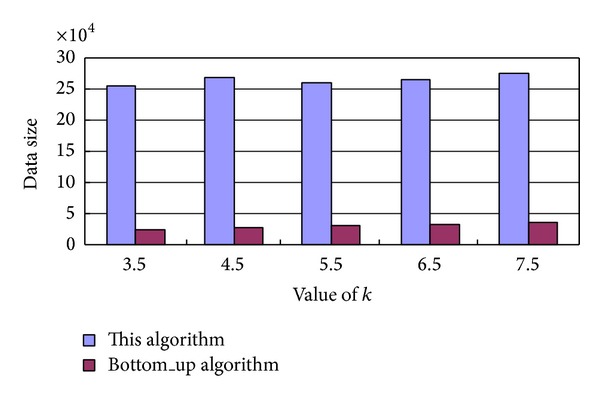
Size of processed anonymous data for our algorithm and the Bottom_up algorithm.

Figure 6 gives the size of the processed anonymous data, in which our algorithm and the Bottom_up algorithm are executed in 20 minutes.

We can see from Figure 6 that, if there are adequate location service requests, our presented algorithm executes multiple parallel threads to search quickly the candidate anonymous sets and it can process more location service requests than the Bottom_up algorithm. That is to say, our algorithm can offer simultaneously services for more users.

## 5. Conclusion

The main contribution of this paper is to establish the location request queues according to the location indexes of the users such that the size of searching information can be remarkably reduced when the anonymous operations are executed and the selection of the anonymous sets on the anonymous server can be speeded up by executing multiple threads to search in parallel the candidate anonymous sets. The presented efficient privacy-preserving location-based query algorithm can obtain better location information services. The next work is to integrate the anonymous locations and the trajectory services into cartographic information and history data to develop the trajectory privacy-preserving method in the distributed networks.

## Figures and Tables

**Table 1:** Experimental parameters.

Average service delay request (second)	Average location precision (mile)	Request amount	Average spatial request (*k*_*s*_)	Average temporal request (*k*_*t*_)	Minimum radius *R*_min⁡_ in average anonymous area (mile)	Maximum radius *R*_max⁡_ in average anonymous area (mile)
1	50.03	466034	4.49	4.50	274.94	637.11
1	50.04	503432	3.50	3.50	275.43	637.43
1	50.08	453293	2.50	2.50	275.10	636.67
5	50.06	457712	4.50	4.50	275.08	637.25
5	49.98	446796	3.50	3.50	275.19	636.99
5	50.01	442778	2.50	2.50	275.12	637.86
10	50.08	456924	4.50	4.50	274.79	637.74
10	49.93	697428	3.50	3.49	275.01	637.54
10	49.98	681940	2.50	2.49	274.84	637.43
10	50.00	493648	2.50	2.50	525.21	1263.51
10	49.97	455932	2.49	2.50	774.50	1387.06
20	49.97	448366	2.50	2.51	275.27	637.64
30	50.03	472418	2.50	2.50	275.37	637.87
